# Stress on the Endoplasmic Reticulum Impairs the Photosynthetic Efficiency of Chlamydomonas

**DOI:** 10.3390/ijms252413304

**Published:** 2024-12-11

**Authors:** Sa Chen, Shuyu Li, Shiyuan Qian, Jiale Xing, Jingjing Liao, Zhifu Guo

**Affiliations:** 1 Key Laboratory of Agricultural Biotechnology of Liaoning Province, College of Biosciences and Biotechnology, Shenyang Agricultural University, Shenyang 110161, China; chensa6628@163.com; 2 State Key Laboratory for Quality Ensurance and Sustainable Use of Dao-di Herbs, Artemisinin Research Center, Institute of Chinese Materia Medica, China Academy of Chinese Medical Sciences, Beijing 100700, China; 18637836720@163.com (S.L.); njucmshiyuanqian@163.com (S.Q.); xjl_6971629@163.com (J.X.)

**Keywords:** endoplasmic reticulum stress, chloroplast, photosynthesis, chlamydomonas

## Abstract

Stress on the Endoplasmic reticulum (ER) can severely disrupt cellular function by impairing protein folding and post-translational modifications, thereby leading to the accumulation of poor-quality proteins. However, research on its impact on photosynthesis remains limited. In this study, we investigated the impact of ER stress on the photosynthetic efficiency of Chlamydomonas reinhardtii using pharmacological inducers, tunicamycin (TM) and brefeldin A (BFA), which specifically target the ER. Our measurements of photosynthetic parameters showed that these ER stress-inducing compounds caused a significant decline in photosynthetic efficiency. A proteomic analysis confirmed that TM and BFA effectively induce ER stress, as evidenced by the upregulation of ER stress-related proteins. Furthermore, we observed a widespread downregulation of photosynthesis-related proteins, which is consistent with the results obtained from our measurements of photosynthetic parameters. These findings suggest that the stress on ER has a profound impact on chloroplast function, disrupting photosynthetic processes. This study highlights the critical interdependence between the ER and chloroplasts, and it underscores the broader implications of ER stress on the cellular metabolism and energy efficiency of photosynthetic organisms.

## 1. Introduction

Photosynthesis is the fundamental process by which plants convert sunlight into chemical energy, essential for their growth and survival, ultimately supporting the entire food chain [1]. Photosynthesis occurs in plants and algae within special structures called chloroplasts. Chloroplasts are thought to have originated from cyanobacteria-like organisms that lived independently before being engulfed by a eukaryotic cell through a process known as endocytosis. Over time, both the host and the engulfed organism adapted to live together in a symbiotic relationship, with changes such as gene transfer and genome shrinkage. To function properly, chloroplasts must work with other organelles in the cell to adapt to various environmental factors, such as temperature, light, and water availability [2]. For instance, most chloroplast proteins are encoded in the cell nucleus, and chloroplasts communicate with the nucleus through retrograde signaling. They also exchange ATP with mitochondria, which helps cells adapt to environmental factors [3,4,5,6].

In addition to the above interaction, there is evidence indicating significant communication between chloroplasts and the endoplasmic reticulum (ER), which plays a crucial role in lipid and protein synthesis [6,7]. The ER is a vital organelle responsible for protein synthesis, folding, and post-translational modifications. It facilitates proper protein folding with the help of molecular chaperones and initiates key modifications such as N-glycosylation and disulfide bond formation, which are essential for protein stability and function. The ER also plays a critical role in quality control by identifying and degrading misfolded proteins through the ER-associated degradation (ERAD) pathway, maintaining cellular protein homeostasis [8]. Interactions between the ER and chloroplasts are manifested in the following aspects: physical contact and bidirectional lipid exchange between these organelles facilitate the transfer of signaling molecules and lipids, which are essential for preserving cellular homeostasis [9,10,11,12,13]. Furthermore, harsh environmental conditions can generate reactive oxygen species (ROS) in chloroplasts, thereby damaging them and spreading oxidative stress to the ER [14,15,16].

Most proteins in the chloroplast are synthesized in the cytoplasm and imported into the chloroplast through the translocons on the enveloping membrane, and these proteins typically possess distinct chloroplast-targeting signal peptides [17]. However, some chloroplast proteins lack a chloroplast-targeting signal peptide and instead contain signal peptides associated with the secretory pathway [18]. These proteins are synthesized in the ER, where their N-termini undergo glycosylation and are then transported to the chloroplasts via vesicular trafficking [13,19,20]. Although evidence suggests the presence of some N-glycosylated proteins within chloroplasts, the N-glycosylation process itself occurs exclusively in the ER, not within the chloroplasts [21,22,23]. Arabidopsis CAH1 (α-carbonic anhydrase) contains a predicted ER-targeting peptide at its N-terminus and functions within the chloroplast stroma after being transported from the ER. The transport of CAH1 is sensitive to brefeldin A, a vesicular transport inhibitor, which causes its retention in the ER and Golgi-like structures [19,24]. The inhibition of the N-glycosylation of CAH1 appears to have an adverse effect on photosynthesis [25]. Loss of CAH1 and its homolog in Chlamydomonas results in reduced high Ci-affinity [26]. Rice AmyI-1 is another secreted chloroplast glycoprotein. Disruption in the process of ER-to-Golgi vesicle transport inhibits the targeting of AmyI-1 to the plastid. [27]. Similarly, the plastidial N-glycosylated OsNPP1 (nucleotide pyrophosphatase/phosphodiesterase), another chloroplast protein, follows the secretory pathway for its transport. OsNPP1 is characterized by multiple N-glycosylation sites and a cleavable hydrophobic signal sequence [28]. Other family members, such as OsNPP2 and OsNPP6, have also been shown to utilize the secretory pathway for chloroplast targeting [29]. Recently, a MtTP930 was found to recognize cargo proteins such as NPP and CAH1 in the ER and facilitate their transport to the chloroplast through the secretory pathway [20]. These interconnected effects highlight the complexity of protein N-glycosylation and vesicle trafficking processes critical for chloroplast function.

However, research exploring the impact of ER stress on photosynthesis remains limited. The purpose of this work is to investigate the effects of the stressed ER on the functions of chloroplasts in *Chlamydomonas reinhardtii* (referred to as Chlamydomonas throughout), with a specific focus on photosynthetic activity [30,31]. We used two drugs that specifically induce ER stress, tunicamycin (TM) and brefeldin A (BFA). Studies on Chlamydomonas have shown that TM and BFA treatment causes heightened sensitivity in cells lacking IRE1, a key sensor of ER stress [30]. Specifically, TM inhibits the N-glycosylation of glycoproteins, a process that is absent in chloroplasts [32,33]. This inhibition leads to the accumulation of misfolded proteins in the ER [34]. BFA blocks ER protein trafficking to the Golgi and causes protein accumulation in the ER, contributing to ER stress [35]. Additionally, as a control, we examined the chloroplast stress responses in Chlamydomonas induced by metronidazole (MZ) and neutral red (NR) treatments. MZ disrupts photosynthetic electron transport by inhibiting ferredoxin-linked NADP^+^ reduction in Photosystem I. This leads to the accumulation of ROS in chloroplasts and is commonly used in Chlamydomonas as a specific inducer of H_2_O_2_ production in the chloroplast [36,37]. NR, on the other hand, is a type I photosensitizer and photosynthesis inhibitor. It causes light-dependent toxicity by inducing the formation of singlet oxygen. Unlike H_2_O_2_, singlet oxygen has a short diffusion range, leading to precise disruptions confined to the chloroplast [38].

We utilized a combination of omics techniques and physiological measurements to reveal the dynamic changes in the Chlamydomonas proteome and photosynthetic efficiency when the ER undergoes stress. Our findings show that a stressed ER leads to a reduction in photosynthetic activity, highlighting the critical interdependence between the ER and chloroplasts.

## 2. Results

### 2.1. Photosynthetic Activity Is Impaired After Treatment with Drugs That Induce ER Stress

**Figure 1:**
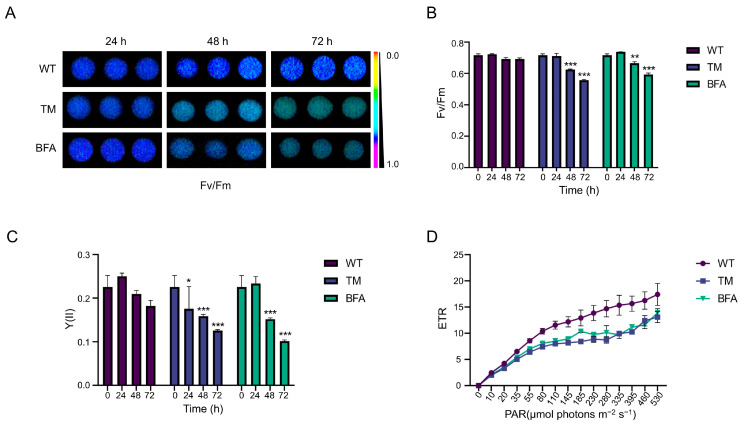
Photosynthetic characteristics of Chlamydomonas treatment with ER stress inducers: (**A**,**B**) Fv/Fm of the Chlamydomonas treatment with the indicated agents. This parameter represents the maximum quantum efficiency of Photosystem II following dark adaptation, which ensures that all reaction centers return to their fully oxidized state. Statistical significance was analyzed using a one-way ANOVA. Significance levels are indicated as *p* < 0.033 (*), *p* < 0.002 (**), and *p* < 0.001 (***). (**C**) Y(II) of the Chlamydomonas treatment with the indicated agent. This parameter reflects the effective quantum yield of PSII under light-adapted conditions, providing a measure of photosynthetic efficiency during active photosynthesis. Statistical significance was analyzed using a one-way ANOVA. Significance levels are indicated as *p* < 0.033 (*), *p* < 0.002 (**), and *p* < 0.001 (***). (**D**) Electron transport rate (ETR) of the Chlamydomonas treatment with the indicated agents. Standard deviations were estimated from three biological replicates.

To investigate whether the ER stress inducers could impair the photosynthetic efficiency of Chlamydomonas, we treated cells with TM and BFA and measured photosynthetic efficiency at different time points (Figure 1). The results revealed that stress on ER significantly affected photosynthetic parameters over time. Fv/Fm, which reflects the maximum quantum efficiency of PSII, remained unchanged at 24 h but showed a marked decline after 48 and 72 h in both TM- and BFA-treated cells compared to the wild type (Figure 1A,B). Similarly, Y(II), a measure of the effective quantum yield of PSII, demonstrated a similar trend (Figure 1C). The electron transport rate (ETR), which evaluates the flow of electrons through the photosynthetic electron transport chain, was also significantly affected. As shown in Figure 1D, ETR values for TM- and BFA-treated cells were consistently lower than those for wild-type cells, particularly at higher light intensities, indicating a disruption in the efficiency of the photosynthetic electron transport chain. These findings collectively demonstrate that ER stress-inducing agents compromise the photosynthetic machinery of chloroplasts, leading to diminished photosynthetic performance over time.

### 2.2. Proteomic Analysis of the Global Protein Expression Profile Under ER Stress Conditions

To understand the effects of the ER stress inducers TM and BFA on cells and how they impact photosynthesis, we performed proteomic analysis on Chlamydomonas treated with these two compounds. A total of 1098 and 1081 proteins were detected in the TM and BFA, respectively, and comprehensive changes in protein expression patterns that represent the biological response to ER stress were found. These proteins were classified according to their expression levels, with log2FoldChange used as a measure to quantify expression changes: significantly increased (log2FoldChange ≥ 1), significantly decreased (log2FoldChange < −1), and unchanged.

Among the total proteins identified, 108 (9.84%) showed a significant increase in expression) and 85 (7.74%) showed a significant decrease in expression after the TM treatment. In the BFA treatment, 82 proteins (7.59% of the total detected proteins) showed a significant increase in expression, while 86 proteins (7.96%) exhibited a significant decrease (Supplemental Data Set).

As a control, we investigated the chloroplast stress responses in Chlamydomonas induced by MZ and NR treatments. In the MZ treatment, a total of 1062 proteins were identified, with 15 proteins (1.41%) significantly upregulated and 21 proteins (1.98%) significantly downregulated. In comparison, the NR treatment revealed 1103 proteins, among which 15 (1.41%) were significantly upregulated, while only 10 (0.91%) were significantly downregulated. Compared with the ER stress results, only one protein, INO1 (Cre03.g180250), was consistently increased in all treatments (Supplemental Data Set). INO1 is essential for myo-inositol synthesis, and it plays a critical role in suppressing cell death [39].

### 2.3. The Features of Protein Expression Patterns Corresponding to ER Stress

**Figure 2:**
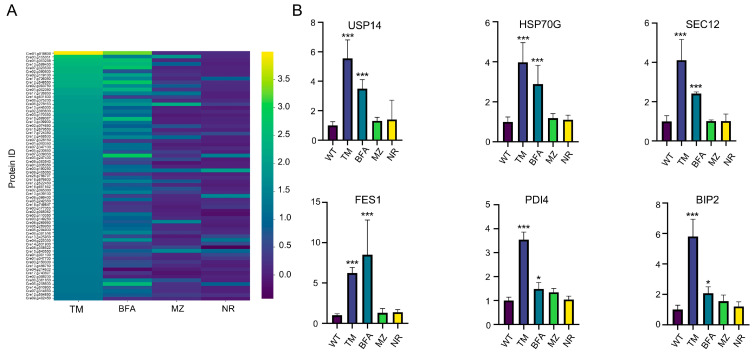
Quantitative analysis of the proteome of Chlamydomonas cells after treatment with different ER or chloroplast stress inducers: (**A**) Heatmap analysis of upregulated proteins (based on TM treatment results) in Chlamydomonas treated with ER stress inducers (TM: 1 μg/mL, BFA: 5 μm) and chloroplast stress inducers (MZ: 2.5 μm, NR: 1.5 mm). Proteins were isolated after 72 h, both with and without the presence of these agents. Differential expression levels are depicted using color coding, as indicated by the scale bar at the bottom of the heatmap. The data are based on three biological replicates for each sample. (**B**) The expression levels of several marker proteins involved in the ER stress response were determined based on proteomics data obtained from three biological replicates. Statistical significance was analyzed using a one-way ANOVA. Significance levels are indicated as *p* < 0.033 (*) and *p* < 0.001 (***).

The proteomic data obtained from the drug treatments revealed distinct trends in protein expression between ER stress inducers (TM and BFA) and chloroplast stress inducers (MZ and NR) (Figure 2A). In particular, marker proteins linked to ER stress exhibited elevated expression levels following TM and BFA treatment. These include protein folding-related proteins BIP2, FES1, PDI4, and HSP70G [40,41,42,43]; the ubiquitin-26S proteasome protein USP14 [44,45]; and the protein transport-related protein SEC12 [46] (Figure 2B). The consistent upregulation of these proteins indicates that TM and BFA successfully induced ER stress, validating the experimental approach. It should be noted that these proteins were upregulated following treatment with ER stress inducers but not following treatment with the chloroplast stressors NR and MZ (Figure 2B).

**Figure 3:**
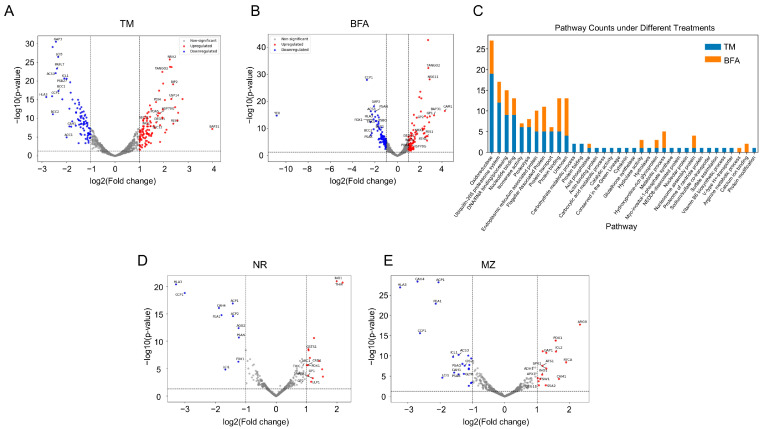
Volcano plot analysis of protein changes and enrichment of significantly upregulated proteins: (**A**,**B**,**D**,**E**) Volcano plots displaying protein expression changes in Chlamydomonas following treatment with TM, BFA, MZ, and NR. The *x*-axis represents the log2 fold change in protein expression, while the *y*-axis represents the −log10 *p*-value. Proteins that significantly changed are marked above the significance threshold. (**C**) Enrichment analysis of significantly upregulated proteins identified in the treatments. The key biological processes, pathways, or cellular components are represented among the upregulated proteins.

In addition to the aforementioned marker proteins, several other key proteins involved in protein folding, degradation, and trafficking pathways in response to ER stress were significantly upregulated in the proteomic analysis of Chlamydomonas treated with TM and BFA (Figure 3A,B, Supplemental Data Set). BAP31 is involved in protein trafficking and quality control, while Nucleoredoxin 2 (NRX2) regulates redox balance and interacts with SEC63 in protein transport. TANGO2 supports Golgi organization and protein transport, and DEG11 is a key protease for protein degradation. The upregulation of these proteins, along with the activation of 26S proteasome subunits, suggests a coordinated response to ER stress through processes like protein turnover, misfolded protein removal, and redox regulation, all essential for maintaining cellular function under stress conditions. TM- and BFA-induced pathways largely overlapped, involving key processes such as oxidoreductase activity, protein turnover, quality control mechanisms, folding, and DNA/RNA binding and processing (Figure 3C). In contrast, the MZ and NR treatments, which target chloroplasts, resulted in the upregulation of proteins involved in oxidative stress responses and metabolic adaptations (Figure 3D,E). Key proteins included those related to vitamin synthesis, glutathione metabolism, and enzymes neutralizing reactive oxygen species (e.g., Glutathione S-transferase GSTS1).

### 2.4. ER Stress Downregulates Photosynthesis-Related Proteins

**Figure 4:**
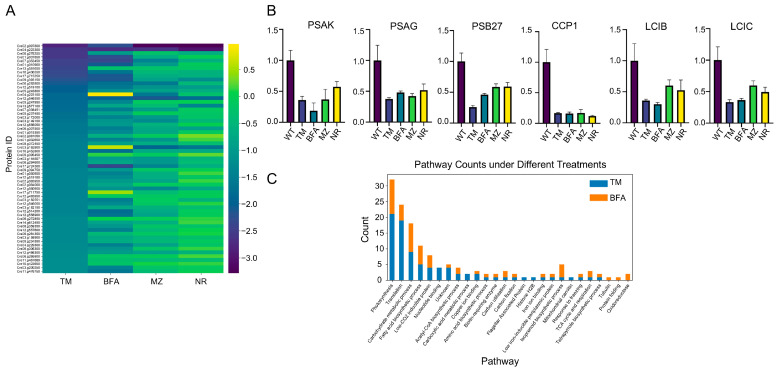
Quantitative analysis of the proteome of Chlamydomonas cells and enrichment of significantly downregulated proteins after treatment with different stress inducers: (**A**) Heatmap analysis of downregulated proteins (based on TM treatment results) in Chlamydomonas treated with ER stress inducers (TM: 1 μg/mL, BFA: 5 μm) and chloroplast stress inducers (MZ: 2.5 μm, NR: 1.5 mm). Proteins were isolated after 72 h, both with and without the presence of these agents. Differential expression levels are depicted using color coding, as indicated by the scale bar at the bottom of the heatmap. The data are based on three biological replicates for each sample. (**B**) The expression levels of photosynthetic and carbon utilization proteins were determined based on proteomics data from three biological replicates. Statistical significance was analyzed using a one-way ANOVA, with all *p*-values < 0.001. (**C**) Enrichment analysis of significantly downregulated proteins across the TM and BFA treatments, highlighting the biological processes, pathways, or cellular components that are overrepresented.

In our analysis of drug treatments, we observed that the ER stress inducers TM and BFA caused a pronounced downregulation of many proteins related to photosynthesis (e.g., PSAK, PSAG, and PSB27) or carbon fixation (e.g., CCP1, LCIB, and LCIC). These proteins exhibited a trend of suppression similar to that observed with the MZ and NR treatments (Figure 4A,B).

Specifically, six Photosystem II subunits—PSBR, PSBQ, PSBO, PSB27, TL19, and PSBP1—were downregulated, along with four Photosystem I subunits—PSAL, PSAK, PSAH, and PSAG; three cytochrome b6f complex proteins—PETO, PETC, and Rieske; and proteins involved in electron transport that may be related to cyclic electron flow—FTRV1 and FNR. Additionally, three proteins involved in the chlorophyll biosynthetic process—CHLI1, CHLP1, and POR1—and one from the light-harvesting complex II LHCB4 were also downregulated. Moreover, proteins related to CO_2_ utilization and fixation, such as LCI5, LCIC, LCI1, LCIB, CAH1, RBCS2, and HLA3, decreased alongside several Calvin cycle enzymes, such as GAP3, FBA3, and SEBP1. Approximately 17 proteins involved in translation (e.g., PRPL7) also showed decreased expression levels, pointing to a slowdown in protein synthesis as a cellular economy measure under stress. Changes in the carbohydrate metabolic process and fatty acid biosynthetic process (e.g., ACC1, ACS3, BCC1, BCC2, and ICL1) indicate alterations in metabolic fluxes to adapt to energy demands under stress (Figure 4C, Supplemental Data Set).

**Figure 5:**
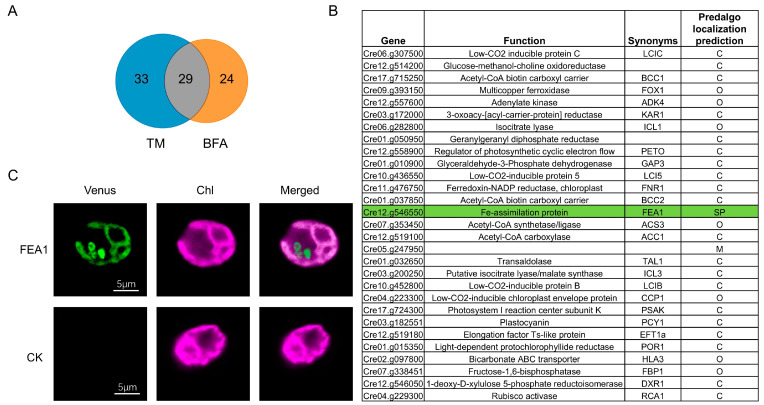
The overlap of differentially expressed proteins (log2FoldChange ≤ −1.2) across the TM and BFA treatments and the localization of FEA1: (**A**) A Venn diagram illustrating the overlap of differentially expressed proteins (log2FoldChange ≤ −1.2) across the TM and BFA treatments. Each section of the diagram represents the proteins uniquely or commonly regulated by the corresponding treatments. (**B**) Detailed descriptions of the 29 commonly regulated proteins, including their Gene IDs, functions, and symbols. The green color represents proteins predicted to be located in the SP. C, chloroplast; O, others; SP; secretory pathway. The protein localization prediction was performed using PredAlgo (http://lobosphaera.ibpc.fr/cgi-bin/predalgodb2.perl?page=main) (accessed on 24 September 2024). (**C**) Confocal microscopy of FEA1-Venus strains, the wild type was used as control (CK).

Similar to TM, BFA treatment also primarily leads to the downregulation of proteins related to photosynthesis and carbon metabolism. They shared 29 downregulated proteins. Almost all of these proteins are involved in photosynthesis, and carbon utilization (Figure 5A,B).

As mentioned above, glycosylated proteins that are transported from the ER to the chloroplast typically have a signal peptide for the secretory pathway. Therefore, we predicted the localization of the 29 downregulated proteins shared by TM and BFA treatments. Most of these proteins either contain a transit peptide for chloroplast targeting or lack a clear signal peptide, with the exception of one protein, FEA1, which is associated with iron absorption and utilization. The prediction suggests that FEA1 does not contain a chloroplast-targeting transit peptide but contains a signal for the secretory pathway (Figure 5B). Localization experiments of this protein show that it is found in structures similar to the secretory pathway; however, it also exhibits clear localization within the chloroplast (Figure 5C). These results suggest that FEA1 may have dual localization; however, whether it undergoes glycosylation in ER and is transported to the chloroplast via the secretory pathway still requires further experimental evidence.

## 3. Discussion

In this work, we studied the coordination between the ER and chloroplasts for maintaining cellular homeostasis and optimizing photosynthetic efficiency [47,48]. Previous studies indicated that stress on ER significantly inhibits the growth of Chlamydomonas [30]; however, these studies did not thoroughly examine the specific effects of ER stress on photosynthesis.

In our study, we selected four compounds: TM and BFA as ER stress inducers, and MZ and NR as chloroplast stress inducers. TM specifically disrupts the N-glycosylation process [49,50], while BFA interferes with ER-to-Golgi COP-I-coated vesicle transport [20,51]. Glycosylation and COP-I-coated vesicle-mediated transport processes are widely accepted to be absent in chloroplasts, as no related enzymes or COP-I-coated vesicle-forming components have been identified, and glycoproteins cannot be expressed in chloroplasts in their glycosylated form [21,22,23,52,53,54,55,56,57]. In our work, the proteomic data comparison treatment with these compounds shows that, unlike chloroplast stress inducers, both TM and BFA impose significant stress on the ER. This stress activates a coordinated network of signaling pathways aimed at mitigating the damage and restoring cellular homeostasis.

Photosynthetic activity, as assessed via the maximum (Fv/Fm) and effective (Y(II)) quantum yields of Photosystem II, was significantly reduced following ER stress. This decline in photosynthetic efficiency was further supported by decreased electron transport rates (ETRs), indicating a compromised photosynthetic apparatus. These findings suggest that the stress on the ER not only disrupts the ER itself but also has a detrimental effect on chloroplast function, ultimately impairing photosynthesis. A proteomic analysis provided deeper insights into the molecular responses to ER stress. Treatment with TM and BFA led to significant alterations in the expression of numerous proteins involved in various cellular processes. A substantial number of proteins associated with photosynthesis were downregulated. This included the subunits of Photosystems I and II, the cytochrome b6f complex, and proteins involved in electron transport and chlorophyll biosynthesis. The downregulation of these critical photosynthetic proteins suggests that the disruption of ER-related processes, including N-glycosylation and vesicle traffic, may prevent the proper modification and transport of photosynthesis-related proteins. On the other hand, it could also represent a cellular strategy to reduce the synthesis of these proteins and thereby limit photosynthetic activity as a response to chloroplast damage.

Certain proteins, including CAH1, OsNPP1, OsNPP2, OsNPP6, and AmyI-1, lack chloroplast-targeting signal peptides; therefore, they do not enter the chloroplast via the classic TIC-TOC translocon system. However, these proteins contain signal peptides for the secretory pathway, and they undergo glycosylation in the ER and are subsequently transported to the chloroplast via the ER-Golgi vesicle trafficking pathway [19,20,27,28,29,52,58,59]. Proteomic studies on glycosylated proteins have identified several photosynthesis-related proteins, although it remains to be determined whether these proteins also follow the secretory pathway to reach the chloroplast [60]. Our study also identified a candidate protein, FEA1, which has a secretory pathway signal peptide and is localized both in vesicles and in the chloroplast. FEA1 plays a key role in iron absorption and utilization, and since iron is essential for the function of electron transport complexes such as PSI, PSII, the cytochrome b6f complex, and ferredoxins, its reduction would impair these processes [61,62,63]. Consequently, a decrease in FEA1 would disrupt iron utilization, leading to a significant impairment in photosynthesis. This gene has a high likelihood (85%) of being involved in photosynthesis, further supporting its critical role in the process [64]. Whether FEA1, like the other proteins mentioned, undergoes glycosylation and is transported to the chloroplast via the vesicle trafficking pathway still requires further experimental validation.

**Figure 6:**
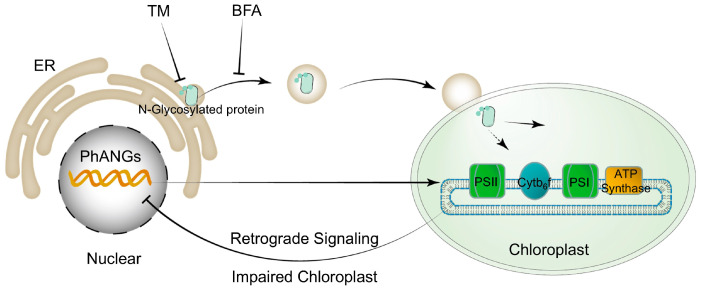
Proposed mechanisms through which ER stress impairs chloroplast functionality and photosynthesis. Stress on ER induced by TM and BFA can interfere with the proper N-glycosylation and vesicular transport of key chloroplast proteins, some of which may be directly involved in photosynthetic processes. This impairment in protein maturation and trafficking can hinder their proper functioning, ultimately decreasing the efficiency of photosynthesis. Additionally, chloroplast damage induced by ER stress may trigger retrograde signaling pathways aimed at protecting the cell from further harm. These signaling pathways often result in the downregulation of nuclear-encoded genes that are essential for chloroplast function, particularly those involved in photosynthesis (PhANGs).

Based on these findings, stress on ER appears to impact chloroplast functionality and photosynthesis through two potential mechanisms (Figure 6). First, ER stress inducers may disrupt the N-glycosylation and vesicle-mediated transport of essential chloroplast proteins. These proteins may include those directly related to photosynthesis, and blocking this pathway could prevent these proteins from functioning properly, leading to a reduction in photosynthetic efficiency. Second, chloroplast damage resulting from stress on the ER might activate retrograde signaling pathways that help mitigate further harm. These pathways could suppress the expression of nuclear-encoded chloroplast genes, particularly those related to photosynthesis (PhANGs) while increasing the expression of stress-responsive genes [65,66]. This coordinated response reduces photosynthetic activity to limit additional damage and enhances the cell’s ability to adapt and survive under stress conditions.

In conclusion, this study underscores the importance of understanding the interconnected nature of cellular organelles and their collective response to stress conditions. ER stress leads to reduced photosynthetic activity and impaired energy production. The extensive proteomic changes observed in response to ER stress reflect the cellular adaptations aimed at mitigating stress-induced damage and preserving homeofstasis. Further research is needed to elucidate the mechanisms linking the ER and photosynthesis, including which photosynthesis-related proteins are transported via the ER-Golgi pathway and the specific role of N-glycosylation in photosynthesis.

## 4. Methods and Materials

### 4.1. Algal Strains and Culture Conditions

Chlamydomonas reinhardtii CC137(mt-) were obtained from the Chlamydomonas Resource Center (https://www.chlamycollection.org/) (accessed on 11 August 2024). The algal cells were cultured in a TAP medium at 25 °C under continuous cool-white, fluorescent light (75 μmol photons m^−2^ s^−1^). The TAP medium used NH_4_Cl as the nitrogen source, and it was prepared following a previously described method [67].

### 4.2. TM, BFA, NR, and MZ Treatments

Cell cultures were kept growing in the exponential phase and diluted to 5 × 10^5^ cells/mL in TAP before drug treatment. The Chlamydomonas cells were treated with 1 μg/mL tunicamycin (TM; Sigma-Aldrich, St. Louis, MO, USA, T7765) diluted from a 5 mg/mL stock in 0.05 N NaOH, 5 μm/mL brefeldin A (BFA; MCE, Shanghai, China, 20350-15-6) diluted from a 10 mm stock in dimethyl sulfoxide (DMSO), 2.5 μm neutral red (NR; BBI, Beijing, China, 553-24-2) diluted from a 1 mm stock in water, and a stock solution of 1.5 mm metronidazole (MZ; Leyan, Beijing, China, 443-48-1) prepared by autoclaving in a TAP medium.

### 4.3. Proteomic Analysis

For a proteomic analysis, we used a previously described method with several modifications [68,69]. The samples treated with different drugs for 72 h were collected and centrifuged for 2 min at 2000× *g*. The supernatant was discarded, and the pellets were resuspended in cell lysis buffer (25 mm Tris-HCl, pH 8.0, 1% SDS, and 25 mm EDTA). A protease inhibitor cocktail and PMSF were added to prevent protein degradation. The mixture was heated at 100 °C for 2 min, followed by centrifugation for 2 min at 2000× *g* to remove cell debris. The supernatant was collected and quantified using a bicinchoninic acid (BCA) assay. The proteins were then reduced with 10 mm dithiothreitol (DTT) and alkylated with 20 mm iodoacetamide (IAA). Subsequently, methanol/chloroform/water (4:3:1) was added to precipitate the proteins. The precipitated proteins were redissolved in 100 μL of 200 mm EPPS buffer (pH 8.5). Lysates containing 100 μg of protein for each sample were digested with sequencing-grade trypsin at 37 °C overnight, desalted using Oasis HLB Extraction Cartridges, completely dried, and redissolved in 100 mm TEAB. The samples were labeled with TMT 10-plex isobaric label reagent and incubated for 2 h in the dark. The labeling was quenched using 1 M Tris buffer (pH7.4), then acidified using 0.1% FA, pooled, and desalted, repeating the previous process (wash solvent: 0.1% FA; elution solvent: 60% ACN in 0.1% FA). Dried peptide samples were resolved in 0.1 formic acid and 1% acetonitrile. Before sample loading, the peptide concentrations were measured (Pierce Quantitative Colorimetric Peptide Assay (no. 23275, Thermo Scientific, Waltham, MA, USA)). All proteomic samples were analyzed with an Orbitrap Fusion Lumos mass spectrometer (Thermo Fisher Scientific, Waltham, MA, USA) coupled with an Ultimate 3000 nani-LC system. The MS raw files were processed and analyzed using Proteome Discoverer 2.4 (Thermo Fisher Scientific) in conjunction with the Mascot search engine (version 2.6.1).

### 4.4. Photosynthetic Activity Determination

Chlamydomonas cells were incubated in the dark for at least 15–20 min with vigorous shaking before measurements. For light–response curve analyses and fluorescence imaging, an IMAGING-PAM system (Walz) was employed. The details have been described previously [70].

### 4.5. Construction and Confocal Microscopy of FEA1-Venus Strains

The gDNA of FEA1 was amplified and ligated into the HpaI-digested PMO449 vector. Nuclear transformation of the CC137 strain with pMO449-FEA1-VENUS was carried out using electroporation as previously described [71]. The resulting transformants were recovered in 1 mL of fresh TAP medium supplemented with 40 mm sucrose for 10 min, followed by plating on TAP medium containing 10 µg/mL paromomycin and incubated at 25 °C under constant light (60 µmol photons m^−2^ s^−1^). After one week, single colonies were picked. Laser scanning confocal microscopy was performed using a Leica TCS SP8. Cells were cultured under light conditions (60 µmol photons m^−2^ s^−1^). For Venus fluorescence, excitation and emission wavelengths were set to 514 nm and 529 nm, respectively. For chlorophyll fluorescence, excitation and emission wavelengths were set to 561 nm and 685 nm, respectively.

## Data Availability

Data are contained within the article.

## Supplementary Materials

The following supporting information can be downloaded at: https://www.mdpi.com/article/10.3390/ijms252413304/s1.

## References

[1] Hohmann-Marriott M.F., Blankenship R.E. (2011). Evolution of photosynthesis. Annu. Rev. Plant Biol..

[2] Aro E.-M., Andersson B. (2006). Regulation of Photosynthesis.

[3] Mostowska A. (1997). Environmental factors affecting chloroplasts. Handbook of Photosynthesis.

[4] Pfalz J., Liebers M., Hirth M., Grübler B., Holtzegel U., Schröter Y., Dietzel L., Pfannschmidt T. (2012). Environmental control of plant nuclear gene expression by chloroplast redox signals. Front. Plant Sci..

[5] Liu J.-X., Howell S.H. (2010). Endoplasmic reticulum protein quality control and its relationship to environmental stress responses in plants. Plant Cell.

[6] Howell S.H. (2013). Endoplasmic reticulum stress responses in plants. Annu. Rev. Plant Biol..

[7] Jacquemyn J., Cascalho A., Goodchild R.E. (2017). The ins and outs of endoplasmic reticulum-controlled lipid biosynthesis. EMBO Rep..

[8] Iurlaro R., Muñoz-Pinedo C. (2016). Cell death induced by endoplasmic reticulum stress. FEBS J..

[9] Hölzl G., Dörmann P. (2019). Chloroplast lipids and their biosynthesis. Annu. Rev. Plant Biol..

[10] Wang Z., Benning C. (2012). Chloroplast lipid synthesis and lipid trafficking through ER–plastid membrane contact sites. Biochem. Sos. Trans..

[11] Siebertz H.P., Heinz E., Linscheid M., Joyard J., DOUCE R. (1979). Characterization of lipids from chloroplast envelopes. Eur. J. Biochem..

[12] Cook R., Lupette J., Benning C. (2021). The role of chloroplast membrane lipid metabolism in plant environmental responses. Cells.

[13] Block M.A., Jouhet J. (2015). Lipid trafficking at endoplasmic reticulum–chloroplast membrane contact sites. Curr. Opin. Cell Biol..

[14] Foyer C.H., Hanke G. (2022). ROS production and signalling in chloroplasts: Cornerstones and evolving concepts. Plant J..

[15] Li M., Kim C. (2022). Chloroplast ROS and stress signaling. Plant Commun..

[16] Ozgur R., Uzilday B., Sekmen A.H., Turkan I. (2015). The effects of induced production of reactive oxygen species in organelles on endoplasmic reticulum stress and on the unfolded protein response in Arabidopsis. Ann. Bot..

[17] Soll J., Schleiff E. (2004). Protein import into chloroplasts. Nat. Rev. Mol. Cell Biol..

[18] Kleffmann T., Russenberger D., von Zychlinski A., Christopher W., Sjolander K., Gruissem W., Baginsky S. (2004). The *Arabidopsis thaliana* chloroplast proteome reveals pathway abundance and novel protein functions. Curr. Biol..

[19] Villarejo A., Burén S., Larsson S., Déjardin A., Monné M., Rudhe C., Karlsson J., Jansson S., Lerouge P., Rolland N. (2005). Evidence for a protein transported through the secretory pathway en route to the higher plant chloroplast. Nat. Cell Biol..

[20] Liu J., Chen H., Liu L., Meng X., Liu Q., Ye Q., Wen J., Wang T., Dong J. (2024). A cargo sorting receptor mediates chloroplast protein trafficking through the secretory pathway. Plant Cell.

[21] Grabsztunowicz M., Koskela M.M., Mulo P. (2017). Post-translational Modifications in Regulation of Chloroplast Function: Recent Advances. Front. Plant Sci..

[22] Dance A. (2010). From pond scum to pharmacy shelf. Nat. Med..

[23] Almaraz-Delgado A.L., Flores-Uribe J., Perez-Espana V.H., Salgado-Manjarrez E., Badillo-Corona J.A. (2014). Production of therapeutic proteins in the chloroplast of *Chlamydomonas reinhardtii*. AMB Express.

[24] Burén S., Ortega-Villasante C., Blanco-Rivero A., Martínez-Bernardini A., Shutova T., Bako L., Villarejo A., Samuelsson G. (2010). N-glycosylation is Required for Trafficking and Activity of a Chloroplast-Localized Carbonic Anhydrase (CAH1) in Arabidopsis thaliana. https://www.diva-portal.org/smash/record.jsf?pid=diva2%3A284336&dswid=6218.

[25] Jiao Q.-S., Niu G.-T., Wang F.-F., Dong J.-Y., Chen T.-S., Zhou C.-F., Hong Z. (2020). N-glycosylation regulates photosynthetic efficiency of *Arabidopsis thaliana*. Photosynthetica.

[26] Shimamura D., Ikeuchi T., Matsuda A., Tsuji Y., Fukuzawa H., Mochida K., Yamano T. (2024). Periplasmic carbonic anhydrase CAH1 contributes to high inorganic carbon affinity in *Chlamydomonas reinhardtii*. Plant Physiol..

[27] Kitajima A., Asatsuma S., Okada H., Hamada Y., Kaneko K., Nanjo Y., Kawagoe Y., Toyooka K., Matsuoka K., Takeuchi M. (2009). The rice alpha-amylase glycoprotein is targeted from the Golgi apparatus through the secretory pathway to the plastids. Plant Cell.

[28] Nanjo Y., Oka H., Ikarashi N., Kaneko K., Kitajima A., Mitsui T., Munoz F.J., Rodriguez-Lopez M., Baroja-Fernandez E., Pozueta-Romero J. (2006). Rice plastidial N-glycosylated nucleotide pyrophosphatase/phosphodiesterase is transported from the ER-golgi to the chloroplast through the secretory pathway. Plant Cell.

[29] Kaneko K., Takamatsu T., Inomata T., Oikawa K., Itoh K., Hirose K., Amano M., Nishimura S., Toyooka K., Matsuoka K. (2016). N-Glycomic and Microscopic Subcellular Localization Analyses of NPP1, 2 and 6 Strongly Indicate that trans-Golgi Compartments Participate in the Golgi to Plastid Traffic of Nucleotide Pyrophosphatase/Phosphodiesterases in Rice. Plant Cell Physiol..

[30] Yamaoka Y., Choi B.Y., Kim H., Shin S., Kim Y., Jang S., Song W.Y., Cho C.H., Yoon H.S., Kohno K. (2018). Identification and functional study of the endoplasmic reticulum stress sensor IRE 1 in *Chlamydomonas reinhardtii*. Plant J..

[31] Yamaoka Y., Shin S., Choi B.Y., Kim H., Jang S., Kajikawa M., Yamano T., Kong F., Légeret B., Fukuzawa H. (2019). The bZIP1 transcription factor regulates lipid remodeling and contributes to ER stress management in *Chlamydomonas reinhardtii*. Plant Cell.

[32] Muthamilselvan T., Kim J.S., Cheong G., Hwang I. (2019). Production of recombinant proteins through sequestration in chloroplasts: A strategy based on nuclear transformation and post-translational protein import. Plant Cell Rep..

[33] Buren S., Ortega-Villasante C., Blanco-Rivero A., Martinez-Bernardini A., Shutova T., Shevela D., Messinger J., Bako L., Villarejo A., Samuelsson G. (2011). Importance of post-translational modifications for functionality of a chloroplast-localized carbonic anhydrase (CAH1) in *Arabidopsis thaliana*. PLoS ONE.

[34] Bull V.H., Thiede B. (2012). Proteome analysis of tunicamycin-induced ER stress. Electrophoresis.

[35] Je S., Choi B.Y., Kim E., Kim K., Lee Y., Yamaoka Y. (2024). Sterol Biosynthesis Contributes to Brefeldin-A-Induced Endoplasmic Reticulum Stress Resistance in *Chlamydomonas reinhardtii*. Plant Cell Physiol..

[36] Edwards D.I., Mathison G.E., Platt D.J. (1974). Metronidazole—An antimicrobial drug which inhibits photosynthesis. Z. Pflanzenphysiol..

[37] Perlaza K., Toutkoushian H., Boone M., Lam M., Iwai M., Jonikas M.C., Walter P., Ramundo S. (2019). The Mars1 kinase confers photoprotection through signaling in the chloroplast unfolded protein response. eLife.

[38] Fischer B.B., Krieger-Liszkay A., Eggen R.L. (2004). Photosensitizers neutral red (type I) and rose bengal (type II) cause light-dependent toxicity in *Chlamydomonas reinhardtii* and induce the Gpxh gene via increased singlet oxygen formation. Environ. Sci. Technol..

[39] Donahue J.L., Alford S.R., Torabinejad J., Kerwin R.E., Nourbakhsh A., Ray W.K., Hernick M., Huang X., Lyons B.M., Hein P.P. (2010). The *Arabidopsis thaliana* Myo-inositol 1-phosphate synthase1 gene is required for Myo-inositol synthesis and suppression of cell death. Plant Cell.

[40] Noh S.J., Kwon C.S., Oh D.H., Moon J.S., Chung W.I. (2003). Expression of an evolutionarily distinct novel BiP gene during the unfolded protein response in *Arabidopsis thaliana*. Gene.

[41] Cho Y., Kanehara K. (2017). Endoplasmic Reticulum Stress Response in Arabidopsis Roots. Front. Plant Sci..

[42] Gowda N.K., Kandasamy G., Froehlich M.S., Dohmen R.J., Andreasson C. (2013). Hsp70 nucleotide exchange factor Fes1 is essential for ubiquitin-dependent degradation of misfolded cytosolic proteins. Proc. Natl. Acad. Sci. USA.

[43] Okumura M., Noi K., Kanemura S., Kinoshita M., Saio T., Inoue Y., Hikima T., Akiyama S., Ogura T., Inaba K. (2019). Dynamic assembly of protein disulfide isomerase in catalysis of oxidative folding. Nat. Chem. Biol..

[44] Xu D., Shan B., Lee B.H., Zhu K., Zhang T., Sun H., Liu M., Shi L., Liang W., Qian L. (2015). Phosphorylation and activation of ubiquitin-specific protease-14 by Akt regulates the ubiquitin-proteasome system. eLife.

[45] Moghadami A.A., Aboutalebi Vand Beilankouhi E., Kalantary-Charvadeh A., Hamzavi M., Mosayyebi B., Sedghi H., Ghorbani Haghjo A., Nazari Soltan Ahmad S. (2020). Inhibition of USP14 induces ER stress-mediated autophagy without apoptosis in lung cancer cell line A549. Cell Stress Chaperones.

[46] Barlowe C., Schekman R. (1993). SEC12 encodes a guanine-nucleotide-exchange factor essential for transport vesicle budding from the ER. Nature.

[47] Bobik K., Burch-Smith T.M. (2015). Chloroplast signaling within, between and beyond cells. Front. Plant Sci..

[48] He C., Berkowitz O., Hu S., Zhao Y., Qian K., Shou H., Whelan J., Wang Y. (2023). Co-regulation of mitochondrial and chloroplast function: Molecular components and mechanisms. Plant Commun..

[49] Yoo J., Mashalidis E.H., Kuk A.C.Y., Yamamoto K., Kaeser B., Ichikawa S., Lee S.Y. (2018). GlcNAc-1-P-transferase-tunicamycin complex structure reveals basis for inhibition of N-glycosylation. Nat. Struct. Mol. Biol..

[50] Ceriotti A., Duranti M., Bollini R. (1998). Effects of N-glycosylation on the folding and structure of plant proteins. J. Exp. Bot..

[51] Barzilay E., Ben-Califa N., Hirschberg K., Neumann D. (2005). Uncoupling of brefeldin a-mediated coatomer protein complex-I dissociation from Golgi redistribution. Traffic.

[52] Ahmad N., Mehmood M.A., Malik S. (2020). Recombinant Protein Production in Microalgae: Emerging Trends. Protein Pept. Lett..

[53] Shamriz S., Ofoghi H. (2019). Expression of Recombinant PfCelTOS Antigen in the Chloroplast of *Chlamydomonas reinhardtii* and its Potential Use in Detection of Malaria. Mol. Biotechnol..

[54] Daniell H., Singh N.D., Mason H., Streatfield S.J. (2009). Plant-made vaccine antigens and biopharmaceuticals. Trends Plant Sci..

[55] Karim S., Aronsson H. (2014). The puzzle of chloroplast vesicle transport—Involvement of GTPases. Front. Plant Sci..

[56] Lindquist E., Alezzawi M., Aronsson H. (2014). Bioinformatic indications that COPI- and clathrin-based transport systems are not present in chloroplasts: An Arabidopsis model. PLoS ONE.

[57] Mathieu-Rivet E., Kiefer-Meyer M.C., Vanier G., Ovide C., Burel C., Lerouge P., Bardor M. (2014). Protein N-glycosylation in eukaryotic microalgae and its impact on the production of nuclear expressed biopharmaceuticals. Front. Plant Sci..

[58] Chen M.H., Huang L.F., Li H.M., Chen Y.R., Yu S.M. (2004). Signal peptide-dependent targeting of a rice alpha-amylase and cargo proteins to plastids and extracellular compartments of plant cells. Plant Physiol..

[59] Akmal M.A., Rasool N., Khan Y.D. (2017). Prediction of N-linked glycosylation sites using position relative features and statistical moments. PLoS ONE.

[60] Wang J., Wen H., Li M., Guo T., Chen C. (2020). N-Glycoproteome Reveals That N-Glycosylation Plays Crucial Roles in Photosynthesis and Carbon Metabolism in Young Rice Leaves. J. Plant Biol..

[61] Nam H.I., Shahzad Z., Dorone Y., Clowez S., Zhao K., Bouain N., Lay-Pruitt K.S., Cho H., Rhee S.Y., Rouached H. (2021). Interdependent iron and phosphorus availability controls photosynthesis through retrograde signaling. Nat. Commun..

[62] Briat J.F., Dubos C., Gaymard F. (2015). Iron nutrition, biomass production, and plant product quality. Trends Plant Sci..

[63] Balk J., Pilon M. (2011). Ancient and essential: The assembly of iron-sulfur clusters in plants. Trends Plant Sci..

[64] Fauser F., Vilarrasa-Blasi J., Onishi M., Ramundo S., Patena W., Millican M., Osaki J., Philp C., Nemeth M., Salome P.A. (2022). Systematic characterization of gene function in the photosynthetic alga *Chlamydomonas reinhardtii*. Nat. Genet..

[65] Richter A.S., Nagele T., Grimm B., Kaufmann K., Schroda M., Leister D., Kleine T. (2023). Retrograde signaling in plants: A critical review focusing on the GUN pathway and beyond. Plant Commun..

[66] Loudya N., Barkan A., Lopez-Juez E. (2024). Plastid retrograde signaling: A developmental perspective. Plant Cell.

[67] Gorman D.S., Levine R.P. (1965). Cytochrome f and plastocyanin: Their sequence in the photosynthetic electron transport chain of Chlamydomonas reinhardi. Proc. Natl. Acad. Sci. USA.

[68] Xing J., Pan J., Yi H., Lv K., Gan Q., Wang M., Ge H., Huang X., Huang F., Wang Y. (2022). The plastid-encoded protein Orf2971 is required for protein translocation and chloroplast quality control. Plant Cell.

[69] Lyu H.N., Fu C., Chai X., Gong Z., Zhang J., Wang J., Wang J., Dai L., Xu C. (2023). Systematic thermal analysis of the Arabidopsis proteome: Thermal tolerance, organization, and evolution. Cell Syst..

[70] Xing J., Liu P., Zhao L., Huang F. (2017). Deletion of CGLD1 Impairs PSII and Increases Singlet Oxygen Tolerance of Green Alga *Chlamydomonas reinhardtii*. Front. Plant Sci..

[71] Shimogawara K., Fujiwara S., Grossman A., Usuda H. (1998). High-efficiency transformation of *Chlamydomonas reinhardtii* by electroporation. Genetics.

